# The Culture of Volunteerism: Attitudes and Motivations among Generation 1.5 Former Soviet Union Immigrants versus Native-Born Israelis

**DOI:** 10.3390/ijerph191912783

**Published:** 2022-10-06

**Authors:** Ester Zychlinski, Maya Kagan

**Affiliations:** Department of Social Work, Ariel University, Ariel 40700, Israel

**Keywords:** FSU, volunteerism, Generation 1.5, Israel, motivation for volunteerism

## Abstract

The large Former Soviet Union (FSU) immigration of the 1990s, accounting for approximately 15% of the Jewish population in Israel today, plays a significant role in shaping Israeli society. Volunteering, as part of social citizenship and normative culture, is an important element of acculturation among immigrants. The present study compares volunteering attitudes and motivations among Generation 1.5 FSU immigrants in their third decade in Israel to native-born Israelis (NBIs): 576 participants, 50.2% NBIs and 49.8% FSUs. Generation 1.5 FSU immigrants exhibited less favorable attitudes toward volunteerism than NBIs. FSUs also rated career motivations for volunteerism significantly higher than NBIs, and values significantly lower than NBIs. Significant differences were found between motivations for volunteering among each cohort, separately, as well. In the context of mass immigration, re-socialization regarding volunteering among Generation 1.5 FSU immigrants living 2–3 decades in Israel appears relatively weak, possibly attributable to FSU policy and culture in opposition to independent volunteerism.

## 1. Introduction

A country founded on the principle of an “ingathering of the exiles” from the international Jewish Diaspora, Israel possesses a rich history of immigration and integration into a host country. In particular, the wave of immigration from the Former Soviet Union (FSU) that occurred in the 1990s is considered one of the largest and most significant of all the various immigration waves the country has experienced. Currently comprising approximately 15% of the entire Israel population [1], this immigration influx received a great deal of academic attention from a broad range of perspectives on immigrant acculturation, including explorations of cultural preferences [2], involvement in political life [3], quality of life [4], family division of labor [5], and housing [6]. “Acculturation is the process of cultural change that occurs as a result of contact between members of two or more cultural groups” [7] (p. 568). This multidimensional process encompasses the acquisition of the local language(s) and customs (e.g., norms and values), and identification both with their culture of origin and new host culture [7,8].

Volunteering, as part of social citizenship and normative culture, is an important element of acculturation [8]. However, despite its social significance, immigrant volunteerism has been a relatively neglected topic of investigation. Several studies conducted in this field suggest that for a brief period following their immigration, the volunteerism patterns of immigrants diverge quite strikingly from those of the local populace (evidencing a lower percentage of volunteering) due to cultural factors and initial adjustment challenges [9]. Over the course of time, this disparity diminishes, and the volunteerism rate increases to almost the same level as the host culture at approximately twenty years post-immigration. This phenomenon typically occurs in countries in which the immigrant group does not constitute a critical mass of the population and adjusts more quickly to local volunteerism patterns [10].

The FSU immigration wave into Israel in the 1990s forms a model test case for examining the volunteerism patterns of immigrants two or three decades after their arrival in the country due to three principal factors. The first is that it brought a critical mass of immigrants to the country. The arrival of such a significant number of immigrants (835,000 from the FSU between 1989 and 2000) may have led to a dual assimilation process in which the mutual influence between the majority and minority created hybrid cultures governed by new social norms [11]. The second factor is the FSU immigration wave’s great “human capital” [12] due to its members’ great pride in their country of origin and motivation to preserve their heritage [13]. An immigrant population of this type may lead to a pattern of volunteering that will preserve their heritage/culture and/or spread it to the wider society. Indeed, the thick ethnicity of FSU immigrants and the “reactive ethnicity” (association with one’s ethnicity in reaction to discrimination) of their migrant children produced an emphasis on civic activity. This promoted the development of third-sector organizations dedicated to causes not only important to this population, but also to the general Israeli population. However, the number of activists among Generation 1.5—a term for those who migrated as a child or adolescent and are thereby characterized by the “in-betweenness” of two cultures [11,14] is still small and concentrated in the big cities [15].

The third factor making the FSU immigration wave ideal for exploring its volunteerism pattern is the impact of Soviet socialization mechanisms and social control that influenced the development of modern, secular citizens strongly attached to the values and symbols of their country of origin [16]. Soviet citizens were not encouraged to engage in private volunteerism of their own volition because they were instead subject to compulsory national “voluntarism” [17,18]. This may account for the low rates of volunteerism amongst the FSU immigrants after their migration [19,20], and may also have long-range implications for Israeli society in terms of bilateral socialization processes, assimilation, intergenerational transfer [21], and volunteerism [18].

As pointed out by Remennick [21], the most successful integration process occurred among immigrants from the FSU who arrived in Israel before the age of 30. Since volunteering is part of the national Israeli educational curriculum, it is expected that this cohort would adopt the host country’s volunteerism norms during the acculturation process [8]. Furthermore, as volunteering is an important element in social citizenship and normative culture in Israel, the population who arrived in Israel about twenty or thirty years ago may have a major role in shaping this aspect of Israeli society, mainly due to its role in the transfer of norms to the second generation.

In light of the fact that there have been very few studies conducted worldwide on volunteerism among Generation 1.5 two or three decades after immigration, and in light of its social importance for integration into Israeli society, the present study seeks to compare the FSU Generation 1.5 with the native-born Jewish Israeli (NBI) populace in regard to attitudes toward volunteerism and motivations to volunteer. The research may help in the development of timely, relevant national, state, and local community policies and practices (e.g., programs, incentives) regarding volunteerism within Israel and also among other Western cultures coping with mass immigration waves.

## 2. Volunteerism in Israel

As in many Western countries, Israel has experienced a clear trend toward individualism in recent decades [22,23]. This is despite a parallel maintenance in the collectivist values rooted in Jewish tradition and socialist ideology that formed the basis upon which the State of Israel was established, in addition to the strong sense of national unity and solidarity created by the shadow of war and security threats [24,25]. This duality is reflected in studies of organized volunteerism and charitable institutions in Israel [25] that have identified a number of national “developmental stages” that oscillate in their emphasis on different values. In the pre-State era, organized volunteerism was characterized by a sectorial and pioneering spirit, its goal being to establish the State and promote a common language. During the next period (1948 to the 1970s), the State sought to limit volunteer organizations to those devoted to State-building activities (absorption of immigrants, etc.), while the late 1970s to mid-1980s saw volunteerism become more sectorial and individualistic again, serving the various cultures, needs, and interests of Israeli society. From the middle of the 1980s, and more intensely during the 1990s, the Israeli third sector once more gained strength through accelerated proliferation and expansion.

To more formally examine volunteerism dynamics in Israel, a systematic study began in 1997 with the establishment of the Israeli Center for Third Sector Research (ICTR) at Ben-Gurion University of the Negev. Subsequently, the Central Bureau of Statistics (CBS) began collecting precise data relating to volunteerism in Israel in 2002; the statistics it has published to date indicate that the profile of the prototypical Israeli involved in organized volunteer activity closely corresponds to that of the Western volunteer: religious and possessing a higher education and a good income [26]. In contrast to most other Western countries, however, more males than females volunteer in Israel [27,28]. That said, such findings are derived from volunteerism in formal organizations in Israel; however, the rate of informal volunteerism, i.e., independent volunteer activity rather than in the context of an organization, is lower amongst high-income, well-educated, and religious individuals [29].

## 3. Volunteerism amongst FSU Immigrants

The 1990’s wave of FSU immigration is essentially different in character to previous waves. This cohort came to Israel largely due to a “push” to leave the FSU (due to the socio-political climate) rather than a “pull” toward Israeli immigration (e.g., ideological motivation), arrived in much greater numbers, emigrated from a developed country, and brought considerable human and cultural capital that reduced, yet did not completely eliminate, the cultural gap with the local populace [11,18]. They had witnessed dramatic cultural and political upheavals in their country of origin: Glasnost, Perestroika, and the fall of the Soviet Union [30]. The transition from an authoritative to a more democratic system also led to changes in official policies regarding volunteerism. This was exemplified in the shift from a socialistic approach in which “volunteerism” was organized and compulsory, to an individualistic, competitive society [13]. Accordingly, the issue of volunteerism became ignored, since it had acquired a stigma due to its association with political public business. Volunteerism in the FSU thus plummeted, with a different type emerging that, in the absence of effective alternatives, was based primarily on self-help groups [31].

These immigrants’ view of volunteerism was dominated by the Soviet work ethic and allegiance to collective solidarity. These values were enforced by formal sanctions (such as non-promotion) and informal proscription (ridicule and social ostracism) [18] among its citizens who were severely restricted in autonomy. The youth in particular were required to participate in the building of communism via compulsory “volunteer” participation in Party projects. As Sikorskaia notes, “This practice made it possible to get young people acquainted with the relations and obligations of production and to develop and have a real influence on their spiritual and physical abilities” [32] (p. 51). When the Soviet regime fell, the mechanisms undergirding compulsory “volunteerism” also disintegrated, causing the level of volunteerism to drastically decline [33].

Like the majority of the post-Soviet populace, FSU Jews, who had assimilated into their previous society and become an integral part of it, vehemently opposed governmental coercion, including “volunteerism”. Studies relating to volunteering amongst FSU Jews in the post-Soviet period suggest that they tended to distrust charity in general eschewing volunteer activity almost completely [17,34].

The findings of a multidisciplinary infrastructure study examining the integration of FSU immigrants into Israel between 1990 and 2005 [19] indicated that only 6.3% were engaged in some form of volunteerism on a regular basis. The rate was higher in 2014, according to the CBS statistics, reaching 13.2%, in significant contrast to the 22.9% rate amongst secular Jewish Israelis and 32.2% among religious Jewish Israelis (both Jewish populations include the FSU immigrants) [35]. Other countries with FSU immigrants also evince a similar tendency [20]. According to Aleksynska’s [36]) study examining immigrant civic involvement in European countries on the basis of ESS data, although forming the largest immigrant group, FSU immigrants were the least civically involved in all the countries surveyed.

Although FSU immigrants play an active part in economic and political life in Israel, serve in the army, and integrate into the job market and institutes of higher learning, their social ties remain largely within the FSU community. Accordingly, they preserve the social values of their home country across a broad range of fields [13], including volunteerism, according to CBS data [35]. Although their percentage of volunteering is low compared to the population born in Israel, FSU Generation 1.5 has begun to be more civically active both within their community and extending to the broader society via activities such as celebration of Novy God (new year’s celebrations in FSU countries), creation of Facebook community *Generation 1.5*, Russian-language poetry festivals, etc. [15,37].

## 4. Motivations for Volunteerism

According to the National Council for Volunteering in Israel [38], volunteerism involves three central elements: free will, activity on behalf of a third party (not including family members), and absence of financial gain. It reflects the customs embedded in the religion, tradition, and culture to which a person belongs [34] and the socio-political context in which a person acts [39]. Further, as volunteers may have both altruistic and egoistic motivations simultaneously, and their motivations may express cultural and social differences [29], Clary et al. [40] characterized the following six motivations for volunteering:

1. Values: The values and humanistic altruistic care for others for no personal gain [41]. Religion or belief in God tends to serve as a significant motivator, prompting people to act on behalf of society [42,43]. Many volunteers believe in the power of God’s commandments and seek to please God by “walking a godly path” of good, charitable acts [39]. More recently, spiritual motivations independent of formal religion have also been identified as factors that encourage people to take more responsibility for contributing to the good of the whole [44]. Generally speaking, values form a key motivation behind voluntary activity [41].
2. Understanding: The desire to expose oneself to new learning experiences and accumulate knowledge and experience about oneself and/or one’s environment [45].
3. Social: The desire to expand and strengthen one’s social network and establish one’s social status [41]
4. Career: The enabling of inquiries into new career possibilities, and help in acquiring skills to aid in furthering one’s career and career prospects [46].
5. Protection: The protection of one’s ego from negative self-evaluation (e.g., diminishing guilt over being more fortunate than others) and enabling better coping with personal problems (e.g., overcoming a sense of inferiority) [40,41].
6. Enhancement: Enhancing one’s feeling of self-worth, self-esteem, and self-image [40,41].

These motivations may help shed light on the behavioral patterns of FSU immigrants and the anticipated benefits that they feel voluntary activity yields, in comparison with NBIs. The literature shows that while most volunteers are motivated by values/altruism [47], amongst immigrants, and FSU immigrants in particular, career (including present and/or future economic gain) plays an important role [20,32,48,49]. By identifying the motivations behind volunteerism, the measure to which various groups, including immigrants, encourage volunteerism can be deduced. Determining the motivations for volunteering among those who do so and the potential motivations for volunteering among those who do not is essential for promoting volunteerism amongst immigrants in such a way as to best meet their needs [17,34,48].

## 5. Research Objectives

The objectives of this study are to examine the association between country of origin and attitude toward volunteerism, the differences between FSU immigrants and NBIs and between volunteers and non-volunteers with respect to motivations for volunteerism, and the rating of motivations for volunteerism among NBIs and FSU immigrants.

## 6. Methods

### 6.1. Research Population and Sample

The study assesses two populations in Israel: NBIs and Generation 1.5 FSU immigrants from the 1990’s wave of immigration. The sample consisted of 576 participants, of whom 50.2% were NBIs and 49.8% were Generation 1.5 FSU immigrants. The age range of arrival in the country for those from the FSU was 13–19 years old. The mean age of the NBI cohort was 42.88 years (*SD* = 6.2) and the mean age of the FSU cohort was 42.07 years (*SD* = 5.75). Of all respondents, 42.3% had volunteered and 57.7% had not volunteered. When analyzing volunteerism by group, 49.5% of all Israeli born respondents had volunteered versus 35.2% of FSU immigrants (χ^2^_[1]_ = 11.46, *p* < 0.01). Among FSU immigrants, there was no significant difference between the length of time in the country of volunteers (*M* = 17.92, *SD* = 6.09) and that of non-volunteers (*M* = 18.11, *SD* = 5.62) (*t*_[270]_ = −0.25, *p* > 0.05). (For detailed characteristics of the sample, see Table 1).

### 6.2. Data Collection and Processing

The current study received the approval of the researchers’ university institutional ethics committee for non-clinical research in humans. The questionnaire, originally in Hebrew, was translated into Russian by a professional academic translation company and then given to Russian language experts for language and phraseology checking. Following its approval, it was back-translated into Hebrew and compared with the original version in order to prevent significant disparities due to mistranslation. The two versions were found to be completely parallel. Then, the questionnaires were administered to a small sample of respondents (pilot study) to receive an impression of the questions and feedback on the formulation of the questions and their comprehensibility.

Non-random sampling was employed due to the lack of public access to databases that include contact details of the entire NBI and FSU immigrant population. The research assistants employed in the project were instructed to recruit NBI and FSU participants who corresponded demographically with their distribution in the general population (with regard to gender, religiosity, socio-economic status, etc.) and with a wide geographical distribution. Potential participants were recruited at various workplaces and at higher education institutions, as well as at their place of residence and in public places (such as leisure culture venues) and through personal acquaintance and referral to other acquaintances. All questionnaires were completed manually and anonymously by the participants, after signing an informed consent form. After completion, the questionnaires were sealed in envelopes and handed to research assistants, who entered the data into a computer database.

### 6.3. Instruments

Socio-demographic questionnaire: This included items relating to age, gender, country of birth (origin), number of years in the country if not born in Israel, number of years of education, degree of religiosity, and level of income.

Questionnaire regarding volunteerism and attitudes toward volunteerism: Constructed for the purposes of this study, this questionnaire contained two items. To ensure uniform comprehension of the term “volunteerism”, a definition was provided within the questionnaire: “Volunteering is defined as an activity in which individuals invest time and energy (but not money or equipment) of their own free will, with no financial compensation (such as a salary, scholarship, or living expenses), where the beneficiary is a third party (organization and/or people who are not related to the volunteer)”. The first question was: “Did you volunteer for any organization over the past five years? 1 = yes, 2 = no.” The second question was: “Are you generally in favor of volunteerism?”. The respondents were requested to mark the answer that best reflects their attitude toward volunteering. The response options were: 1—I am not in favor of volunteering at all, 2—I am moderately in favor of volunteering, and 3—I am strongly in favor of volunteering.

Volunteer Functions Inventory (VFI) questionnaire [39]: This questionnaire consists of 30 items on a 1–7 Likert scale (1 = completely disagree, 7 = completely agree) relating to the motivations for volunteering. This questionnaire allows identification of which motivations lay behind the participants’ engagement in volunteer activity in practice and the extent to which these influenced those who were not currently engaged in any such activity. Via exploratory and confirmatory factor analyses, Clary et al. [40] identified the existence of subscales representing six primary motivations for volunteering. Sample items for each of the factors: Career—“Volunteering can help me get my foot in the door at a place where I would like to work”; Values—“I am concerned about those less fortunate than myself”; Protection—“Volunteering helps me work through my own personal problems”; Social—“People I know share an interest in community service”; Understanding—“Volunteering allows me to gain a new perspective on things”; and Enhancement—“Volunteering makes me feel better about myself.”.

In the present study, the Cronbach α representing the level of internal consistency reliability for each of six subscales for the entire sample was as follows: career = 0.91, values = 0.81, protection = 0.8, social = 0.78, understanding = 0.87, and enhancement = 0.85. The Cronbach α for assessing the internal consistency reliability for NBIs was as follows: career = 0.88, values = 0.82, protection = 0.79, social = 0.73, understanding = 0.84, and enhancement = 0.84. The Cronbach α for assessing the level of internal consistency reliability for FSU immigrants was as follows: career = 0.93, values = 0.80, protection = 0.82, social = 0.77, understanding = 0.89, and enhancement = 0.87.

## 7. Results

### 7.1. The Association between Origin and Attitude toward Volunteerism

An association was found between origin and attitudes toward volunteerism (χ^2^_[2]_ = 34.86, *p* < 0.001). While 82.7% of NBIs reported a positive attitude toward volunteerism, only 60.6% of the FSU immigrants did so. Ten percent of the NBIs stated that they moderately favored volunteerism, versus 24.7% of the FSU immigrants, and 14.6% of the FSU immigrants declared that they were not in favor of volunteerism at all, versus 7.3% of NBIs.

### 7.2. Motivations for Volunteering

To test whether differences would exist in each of the six identified motivations for volunteering between NBIs and FSU immigrants, whether differences would exist between those actively engaged in volunteering and those not, and whether an interaction would exist between origin and volunteerism, we conducted a two-way ANOVA. In line with the Shapiro-Wilk test, our dependent variables, each of the six motivations for volunteering, were distributed normally for the groups formed by the combination of the origin and volunteerism variables. The Levine test indicated homogeneity of variances across groups for all motivations of volunteering (see Table 2).

1. Career: A main effect for origin was found for the difference between NBIs and FSU immigrants in relation to this motivation (*F*_[1,537]_ = 22.68, *p* < 0.001, partial η^2^ = 0.04). The latter rated this motivation significantly higher than the former. No main effect was found for volunteerism, however, i.e., there was no disparity between volunteers and non-volunteers with respect to career motivation, nor was any interaction effect found between origin and volunteerism.

2. Enhancement: While no main effects were found for country of origin and volunteerism in this context, a significant interaction was found between them (*F*_[1,537]_ = 4.40, *p* < 0.001, partial η^2^ = 0.008). To test the source of this interaction, we conducted a *t*-test for independent samples. The findings indicated that FSU immigrant volunteers rated enhancement higher than their non-volunteering counterparts (*t*_[266]_ = 2.24, *p* < 0.05).

3. Social: A main effect was found for volunteerism (*F*_[1,537]_ = 7.63, *p* < 0.001, partial η^2^ = 0.014). The volunteers rated this motivation significantly higher than the non-volunteers. No main effect was found for origin, i.e., no significant difference was found between NBIs and FSU immigrants, nor was any interaction effect found between origin and volunteerism.

4–5. Protection and Understanding: A two-way ANOVA of each of these motivations revealed no main effects for origin or volunteering, nor did any interaction effect exist between them.

6. Values: While no interaction was found between origin and volunteerism, a main effect was found for each of the variables separately. NBIs rated this motivation higher than the FSU immigrants (*F*_[1,537]_ = 5.72, *p* < 0.001, partial η^2^ = 0.011), and volunteers rated it higher than did non-volunteers (*F*_[1,537]_ = 7.74, *p* < 0.001, partial η^2^ = 0.014).

### 7.3. FSU and NBI Motivation Ranking

As Table 3 indicates, both NBIs and FSU immigrants rated values as the highest motivation. The average rating scores between the two groups nonetheless differed. NBIs rated the six motivations in the following descending order: values, understanding, enhancement, social, protection, and career. FSU immigrants rated them in descending order as: values, understanding, enhancement, career, social, and protection. In order to examine whether there were significant differences in regard to each of the motivations in each group separately, we conducted a General Linear Model for Repeated Measures. The analysis yielded significant findings for both NBIs (*F*_[3.7, 1027.88]_ = 215.25, *p* < 0.001, partial η^2^ = 0.44) and FSU immigrants (*F*_[3.96, 1081.93]_ = 98.71, *p* < 0.001, partial η^2^ = 0.27).

A post hoc test with a Bonferroni correction amongst the NBIs revealed significant differences between most of the ratings, with the exceptions of social and protection motivations that were rated fairly similarly. No significant differences were found amongst the FSUs between career, social, and protection motivations, all of which were rated very similarly, nor did any significant differences exist between understanding and enhancement, despite their significant deviation from the other motivations.

## 8. Discussion

The current study findings are not consistent with previous studies indicating that the lower the age of immigration and the longer the period of residence in the absorbing society, the less the immigrants differ in volunteerism from the local populace [10,34]. Instead, the current findings show that Generation 1.5 FSU immigrants exhibit less positive attitudes toward volunteerism than NBIs. Even after two to three decades in their host country, the Generation 1.5 FSU immigrants appeared to preserve the volunteerism patterns of their country of origin [15,34].

This finding may be explained by the fact that this wave of FSU immigration forms a critical mass in Israel society [11,12], which may mean that volunteering re-socialization, as an acculturation process in the host country, is relatively weak. Our findings seem to challenge Voicu’s [34] expectation that such acculturation processes generally exert a greater influence on immigrants than those they experienced in their country of origin. The acculturation process might also be slower due to the inherent tendency of Generation 1.5 FSU immigrants to preserve their original culture [15]. This explanation is consistent with Tong’s findings [50] indicating the lower tendency of immigrant youth living in immigrant neighborhoods, even those with high levels of human and financial capital, to adopt the accepted local societal patterns of volunteering than those living in non-immigrant neighborhoods.

The differences in the level of volunteering and in attitudes toward volunteerism may also be explained by the variation in religiosity among the FSU population compared to NBIs: the more religious one is, the greater the tendency to volunteer [47]. It may be desirable to examine in further studies the interaction between origin (FSU/ NBI) and religiosity with regard to actual volunteering, attitudes towards volunteering, and motivations for volunteering.

In relation to the motivations for volunteering amongst the two samples, the findings indicate that career motivation, associated with self-support [45] and basic economic welfare [20,48], served as a more significant factor amongst the FSU immigrants than amongst the NBIs. An explanation may lie in the often-challenging economic situation of immigrants in general: the literature indicates that they earn less than those born in the host country, so they may see volunteerism mainly as a way of improving their financial condition [48].

Moreover, career motivation was rated higher by both FSU immigrant volunteers and non-volunteers. Whether actually volunteering or just considering it, career prospects—an essentially economic factor— is a key consideration. Indeed, employment is generally a significant aspect of one’s personal identity, perhaps more so among FSU immigrants in light of the Soviet work ethic. Further, as employment can be critical to the successful integration of immigrants, it may be a significant motivating factor in volunteering [18,48]. The study’s findings reveal that while the two populations both rated values as their highest motivation to volunteer, the average rating of this motivation was lower amongst the FSU Generation 1.5 immigrants. While this may be attributed to the low social value of non-compulsory volunteerism prevalent in the FSU [18,49], another explanation may lie in the fact that some FSU immigrants do not feel that they truly belong to Israeli society and do not fully identify with its values and goals [15]. Thus, they are less likely to be motivated to volunteer because of Israeli values. Moreover, the community’s tendency to maintain a closed structure [13] may contribute to this stance, as articulated by Liu [51] “... a strong sense of place or belonging seemed to be formed based on descent, physical appearances, and values—something that was difficult to change, if not impossible” (p. 34).

In respect to the other motivations, no differences between Generation 1.5 FSU immigrants and NBIs were found. Some results did relate primarily to those engaged in volunteer activities, however. Generation 1.5 FSU immigrants that were engaged in volunteer activity cited enhancement as a more important motivation than non-volunteers. This may be understood in light of findings in other studies suggesting that volunteers in Israel from the FSU in general [18] and FSU Generation 1.5 in specific emphasize the importance of their self- and community empowerment as part of their motivations for volunteering.

Encouraging FSU immigrants to volunteer might enhance feelings of self-empowerment, thereby elevating their sense of self-worth and self-image [40,41]. As such, it could constitute a practical means for promoting the acculturation process among immigrants.

In terms of policy recommendations, countries that are targets of mass immigration, especially from the FSU, should be aware of the unique cultural characteristics of this type of immigration, which may affect the host society. Therefore, it is important to develop civil mechanisms such as voluntary activity that can help with the integration process. To this end, it is important to promote awareness of volunteering among the immigrants by campaigning for their recruitment through the media and social networks in the language of the immigrants. Among other things, it is important to promote effective outreach in absorption centers, as well as in places of residence, work, and leisure of this population. One option may be to promote volunteer activity in schools where their children are enrolled together with NBI children, e.g., family volunteer programs, preferably beginning in kindergarten and primary school, as most parents are accustomed to attending their children’s activities at these ages. This kind of activity could increase bridging social capital between Generation 1.5 FSU immigrants and NBIs, which is critical because bridging social capital is considered indispensable for healthy democratic communities [40] Another way could be to encourage FSU immigrants to participate in volunteer programs in the field of social rights. This type of volunteerism would enhance bonding social capital among FSU immigrants, socializing them with the value of volunteering, and could become an important bridge between them and society at large [51].

Amongst both populations, participant volunteers rated social motivation higher than non-volunteers. This motivation, which pertains to volunteers’ needs for social contact, is a general feature of volunteer activity in which establishing links with others forms a central element [46,52]. Notably, those who volunteer may be more aware of the social benefits of volunteer activity, thus leading them to value social benefits from volunteerism to a higher degree.

### Limitations and Recommendations for Further Study

The study employed a non-random sampling method. Despite the fact that efforts were taken to recruit participants from across the country, the findings do not necessarily represent each cohort’s general population. In subsequent studies, it is advisable to conduct representative samples and qualitative research to address the rating of motivations among both samples.

It would also be worthwhile exploring patterns among FSU Generation 1.5 who immigrated to other countries (e.g., Canada, United States, Germany) to see if there are other cultural variables that may influence the type and priority of motivations for volunteering. Further, examination of intergenerational transmission of such attitudes and motivations and how they manifest in the children of FSU Generation 1.5 born in the host country is also of interest. Scholars should continue to use empirical means to study the factors that help explain immigrant patterns of volunteerism and, in particular, the role it plays in integration and assimilation in the new country and society. The issue of volunteerism in the context of mass immigration has not yet been subject to extensive and in-depth study. Greater understanding of this topic would better equip countries to help immigrants integrate themselves and transform their vast human capital into effective social capital that benefits society as a whole in this era of global migration.

## 9. Conclusions

A healthy democratic society depends heavily on civic participation, in which volunteer activity plays a prominent part [38,53]. The findings of this study show that generation 1.5 FSU immigrants display a lower level of integration in volunteer activity, attitudes, and motivations to volunteering, than do NBIs, even two or three decades after immigration to Israel. Therefore, the host country should intervene and develop specific programs to encourage these immigrants to volunteer on a similar level as NBIs. This will contribute to the well-being of the immigrants and of the society that they have joined.

## Figures and Tables

**Table 1:** Sample characteristics (n = 576).

Characteristics		Values	NBI % (n = 289)	FSU% (n = 287)	Chi Square	*t*-Test
Gender	Volunteers	Male	48.5	49	0.95	
		Female	51.5	51		
	Non-volunteers	Male	32.4	37.9	1.02	
		Female	67.6	62.1		
	Total	Male	39.3	41.1	0.17	
		Female	60.7	58.9		
Religiosity	Volunteers	Ultra-Orthodox	4.4	5.3	38.98 ***	
		Religious	66.2	30.5		
		Traditional	12.5	9.5		
		Secular	16.9	54.7		
	Non-volunteers	Ultra-Orthodox	4.4	0.6	69.87 ***	
		Religious	47.4	17.8		
		Traditional	24.8	10.9		
		Secular	10.9	70.7		
	Total	Ultra-Orthodox	4.5	2.1		
		Religious	55.6	21.7	117.27 ***	
		Traditional	19.8	11.4		
		Secular	20.1	64.8		
Volunteerism	Total	Volunteer	49.5	35.2	11.46 **	
		Not Volunteer	50.5	64.8		
Age M (Sd)	Volunteers		42.68 (6.11)	42.12 (5.58)		0.71
	Non-volunteers		44.32 (6.19)	42.23 (5.84)		1.58
	Total		42.88 (6.2)	42.07 (5.75)		1.62
No. of years of formal education M (Sd)	Volunteers		16.04 (3.15)	16.03 (3.01)		0.03
	Non-volunteers		15.18 (3.31)	15.49 (3.2)		−0.83
	Total		15.58 (3.22)	15.55 (3.38)		0.13
Income M (Sd)	Volunteers		6.87 (2.71)	6.01 (2.71)		2.29 *
	Non-volunteers		6.58 (2.61)	6.2 (2.64)		1.27
	Total		6.73 (2.66)	6.13 (2.66)		2.67 **

* *p* < 0.05, ** *p* < 0.01, *** *p* < 0.001. Note. Income represented on a 1–10 scale, 1 = 2000 NIS or below, each number then marking a 2000 increase.

**Table 2:** Findings of two-way ANOVA regarding motivation for volunteerism according to country of origin and actual volunteer activity (*N* = 576).

Motivation for Volunteerism	Source of Variation	Mean Square	*F*	*P*
Career	Origin	63.63	22.68	0.00
	Volunteerism	3.31	1.12	0.29
	Origin × Volunteerism	0.60	0.21	0.64
Enhancement	Origin	3.26	1.36	0.24
	Volunteerism	3.35	1.39	0.24
	Origin x Volunteerism	10.56	4.40	0.04
Social	Origin	0.41	0.22	0.64
	Volunteerism	14.49	7.63	0.01
	Origin x Volunteerism	3.97	2.09	0.15
Protective	Origin	1.50	0.80	0.37
	Volunteerism	2.23	1.19	0.28
	Origin x Volunteerism	0.07	0.04	0.85
Understanding	Origin	0.34	0.13	0.72
	Volunteerism	2.29	0.87	0.35
	Origin × Volunteerism	2.55	0.97	0.33
Values	Origin	11.40	5.72	0.02
	Volunteerism	15.45	7.74	0.01
	Origin × Volunteerism	1.65	0.83	0.36

**Table 3:** Motivation for volunteerism according to origin and actual volunteer activity (*N* = 576).

		Career *M*(*SD*)	Enhancement *M*(*SD*)	Social *M*(*SD*)	Protective *M*(*SD*)	Understanding *M*(*SD*)	Values *M*(*SD*)
NBIs	Volunteers	2.41(1.37)	3.59(1.59)	3.24(1.44)	3.07(1.29)	3.93(1.60)	5.23(1.40)
	Non-volunteers	2.50(1.56)	3.72(1.48)	3.08(1.33)	2.96(1.34)	3.94(1.50)	5.00(1.41)
	NBI total	2.45(1.47)	3.65(1.53)	3.16(1.39)	3.01(1.31)	3.94(1.55)	5.11(1.41)
FSU immigrants	Volunteers	3.04(1.77)	4.04(1.59)	3.47(1.53)	3.20(1.45)	4.12(1.65)	5.05(1.44)
	Non-volunteers	3.27(1.91)	3.59(1.55)	2.96(1.27)	3.05(1.41)	3.85(1.72)	4.59(1.41)
	FSU total	3.19(1.86)	3.75(1.57)	3.14(1.39)	3.10(1.42)	3.95(1.70)	4.75(1.43)
Total Volunteers		2.67(1.57)	3.78(1.60)	3.33(1.48)	3.12(1.36)	4.01(1.62)	5.16(1.42)
Total Non-volunteers		2.92(1.80)	3.65(1.51)	3.01(1.30)	3.00(1.37)	3.89(1.62)	4.77(1.42)

Note. All comparisons based on a scale of 7 (1 = low, 7 = high).

## Data Availability

The data is available upon request.
